# Identification of BPIFA1/SPLUNC1 as an epithelium-derived smooth muscle relaxing factor

**DOI:** 10.1038/ncomms14118

**Published:** 2017-02-06

**Authors:** Tongde Wu, Julianne Huang, Patrick J. Moore, Michael S. Little, William G. Walton, Robert C. Fellner, Neil E. Alexis, Y. Peter Di, Matthew R. Redinbo, Stephen L. Tilley, Robert Tarran

**Affiliations:** 1 Cystic Fibrosis Center/Marsico Lung Institute, Marsico Hall, 125 Mason Farm Road, University of North Carolina at Chapel Hill, Chapel Hill, North Carolina 27599-7248, USA; 2 Department of Chemistry, Genome Science Building, 250 Bell Tower Road, University of North Carolina at Chapel Hill, Chapel Hill, North Carolina 27599-7248, USA; 3 Center for Environmental Medicine, Asthma, and Lung Biology, US EPA Human Studies Facility, 104 Mason Farm Road, University of North Carolina at Chapel Hill, Chapel Hill, North Carolina 27599-7248, USA; 4 Department of Environmental and Occupational Health, University of Pittsburgh, 331 Bridgeside Point Building, Pittsburgh, Pennsylvania 15260, USA; 5 Department of Cell Biology & Physiology, 5200 Medical Biomolecular Research Building, 111 Mason Farm Road, University of North Carolina at Chapel Hill, Chapel Hill, North Carolina 27599-7248, USA

## Abstract

Asthma is a chronic airway disease characterized by inflammation, mucus hypersecretion and abnormal airway smooth muscle (ASM) contraction. Bacterial permeability family member A1, BPIFA1, is a secreted innate defence protein. Here we show that BPIFA1 levels are reduced in sputum samples from asthmatic patients and that BPIFA1 is secreted basolaterally from healthy, but not asthmatic human bronchial epithelial cultures (HBECs), where it suppresses ASM contractility by binding to and inhibiting the Ca^2+^ influx channel Orai1. We have localized this effect to a specific, C-terminal α-helical region of BPIFA1. Furthermore, tracheas from *Bpifa1*^*−*/*−*^ mice are hypercontractile, and this phenotype is reversed by the addition of recombinant BPIFA1. Our data suggest that BPIFA1 deficiency in asthmatic airways promotes Orai1 hyperactivity, increased ASM contraction and airway hyperresponsiveness. Strategies that target Orai1 or the BPIFA1 deficiency in asthma may lead to novel therapies to treat this disease.

## Abstract (web summary)

Asthma is characterized by abnormal airway hyperresponsiveness. Here the authors identify BPIFA1 as a factor secreted by airway epithelial cells, and show that it regulates contractility of airway smooth muscle cells by binding to and regulating the Orai1 Ca^2+^ channel.

Asthma affects ∼334 million people worldwide, yet little is known regarding the underlying aetiology of the exaggerated airway smooth muscle (ASM) contraction that leads to airway hyperresponsiveness (AHR). More recently, research has focused on the role of airway epithelia in driving asthma. In ASM, the degree of contraction is directly proportional to the cytosolic Ca^2+^ levels^1^,^2^. As such, ASM Ca^2+^ homeostasis is tightly regulated and often involves store-operated Ca^2+^ entry (SOCE), a process whereby stromal interacting molecule 1 (STIM1) relocates/aggregates at the sarcoplasmic reticulum (SR)-plasma membrane junction where it activates Orai1 to allow Ca^2+^ influx, leading to contraction^3^,^4^. SOCE is defective in ASM from asthma patients and in murine asthma models, which show increased Orai1 activity^5^,^6^. For over 30 years, researchers have predicted that an epithelium-derived smooth muscle relaxing factor (EDSMRF) exists in normal subjects where it regulates ASM Ca^2+^ levels, and that EDSMRF is deranged in asthmatics, leading to an exaggerated Ca^2+^ response to agonists such as methacholine (Mch). The existence of an EDSMRF was first proposed when researchers demonstrated that trachea denuded of epithelia induced AHR and that placing epithelia from another animal in the same organ bath reduced contractility^7^. Candidate molecules for EDSMRF have included NO, arachidonic acid metabolites, and cytokines, but all have been ultimately rejected since they do not meet the necessary criteria of (i) being secreted directly into the media and (ii) being modulated by inflammatory mediators^8^. Bacterial permeability family member A1 (BPIFA1), also known as short palate and nasal epithelial clone 1 (SPLUNC1), secreted protein from the upper respiratory tract, LUNg-specific protein-X and nasopharyngeal carcinoma-related protein, is a multi-functional protein that is secreted by airway epithelia and has both antimicrobial activity and regulates ion channels^9^. BPIFA1 production has previously been shown to be inhibited by Th2-type cytokines that are upregulated in asthma, such as IL-13, and in allergic mouse models, suggesting that it plays a role in the allergic response to allergens^10^. However, its effects on ASM have not previously been described. We observed that BPIFA1 protein levels were diminished in sputa from asthma patients. Surprisingly, we found that BPIFA1 was secreted basolaterally from normal human bronchial epithelial cultures (HBECs) and that basolateral BPIFA1 secretions were significantly reduced in asthma-derived HBECs. On the basis of these data, we tested the hypothesis that BPIFA1 was the EDSMRF.

In support of this hypothesis, we found that serosal media from normal but not asthmatic HBECs lowered cytosolic ASM Ca^2+^ levels and prevented ASM contractions. We also found that Bpifa1-deficient (*Bpifa1*^*−*/*−*^) mice exhibited spontaneous AHR and that excised tracheas from *Bpifa1*^*+/+*^ but not *Bpifa1*^*−*/*−*^ mice were capable of reducing ASM contraction. Furthermore, recombinant BPIFA1 blocked Ca^2+^ influx and bound specifically to the plasma membrane Ca^2+^ channel Orai1, leading to Orai1 internalization. Thus, we propose that BPIFA1 (SPLUNC1) is the EDSMRF and it functions by internalizing Orai1.

## Results

### BPIFA1 levels inversely correlate with asthma and AHR

**Figure 1:**
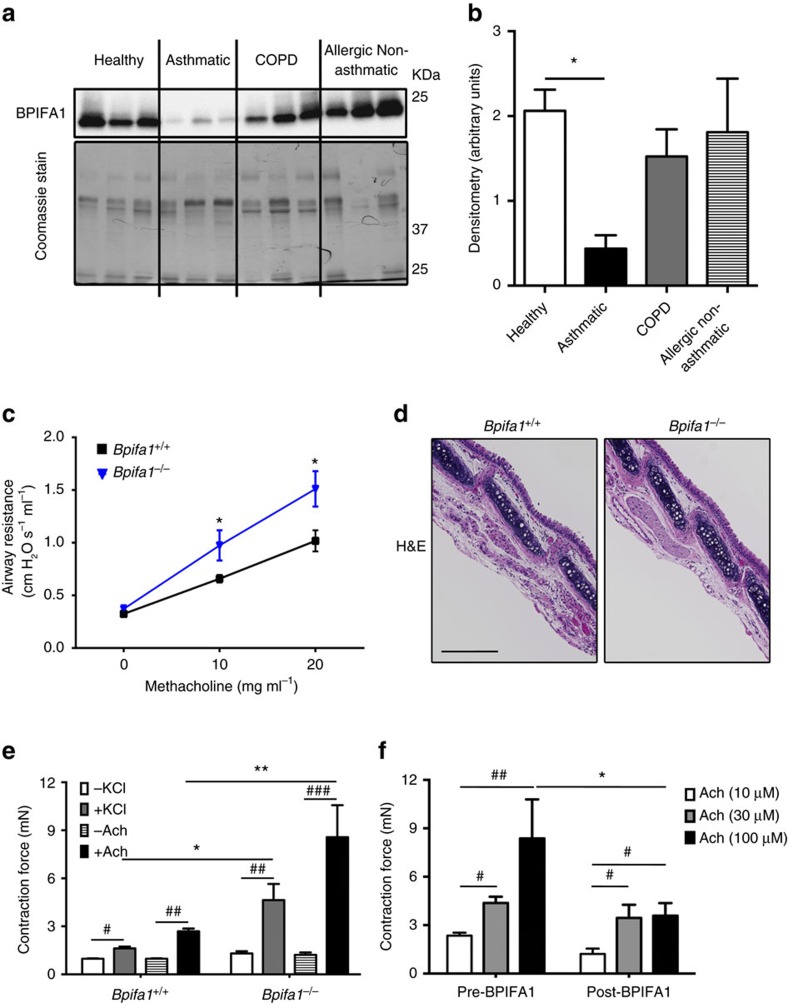
BPIFA1 is diminished in asthmatic airways and is associated with airway hyperresponsiveness (AHR) in mice. Induced sputum was collected from healthy normal controls, asthmatic and chronic obstructive pulmonary disease (COPD) patients, and allergic non-asthmatics. (**a**) Representative immunoblots of BPIFA1 (upper) and coomassie loading control (lower). (**b**) Mean densitometry of BPIFA1 normalized to total protein. (*n*=6 subjects per group). (**c**) Evaluation of peripheral airway resistance by flexiVent after methacholine challenge in *Bpifa1*^*−*/*−*^ and *Bpifa1*^*+/+*^ littermate controls. (**d**) Representative haematoxylin and eosin (H&E) staining of tracheas from *Bpifa1*^*−*/*−*^ and *Bpifa1*^*+/+*^ mice (*n*=3 mice/genotype). Scale bar is 200 μm. (**e**) Tracheal rings (*n*=6 mice per genotype) were extracted from *Bpifa1*^*+/+*^ and *Bpifa1*^*−*/*−*^ mice and mounted onto wire myographs. Contraction was measured under both resting and agonist (KCl and Ach)-induced conditions. (**f**) Contractile force was measured pre- and post-bilateral BPIFA1 addition to the bath 1 h before agonist addition (all *n*=6). Data in **b**,**c**,**e** and **f** are mean±s.e.m. The data were analysed using one-way ANOVA followed by Tukey *post hoc* analysis in **b**, Student's *t*-test in **c** and two-way ANOVA followed by Sidak corrected *post hoc* analysis in **e** and **f**. ^#^and *indicate *P*<0.05, ^##^and **indicate *P*<0.01, ***and ^###^indicates *P*<0.001 different to control.

To investigate the role of BPIFA1 in asthma pathogenesis, we measured sputum BPIFA1 levels in healthy donors, asthmatic patients, chronic obstructive pulmonary disease patients and atopic individuals without asthma, the latter two cohorts serving as disease controls. Immunoblot analyses indicated significantly decreased BPIFA1 protein levels in asthmatic patients' samples compared with the other donors (Fig. 1a,b, Supplementary Table 1). To determine whether a decrease in BPIFA1 levels was associated with abnormal ASM activity, we next tested whether *Bpifa1*^*−*/*−*^ mice exhibit AHR. These mice showed a significant increase in airway resistance following Mch challenge compared with their *Bpifa1*^*+/+*^ littermate controls (Fig. 1c), indicating an inverse correlation between Bpifa1 expression and ASM contraction. Haematoxylin and eosin (H&E) staining and quantification of smooth muscle mass of tracheas from these mice showed no obvious difference in ASM morphology between *Bpifa1*^*+/+*^ and *Bpifa1*^*−*/*−*^ animals (Fig. 1d and Supplementary Fig. 1), suggesting that the increase in airway resistance in *Bpifa1*^*−*/*−*^ mice was not due to ASM hypertrophy. To further study the effect of Bpifa1 on ASM, we excised tracheas from these mice and mounted them on wire myographs to measure contractility *ex vivo*. Tracheal rings from *Bpifa1*^*−*/*−*^ mice showed significant hypercontractility compared with wild-type controls following exposure to acetylcholine (Ach) or KCl (Fig. 1e). Since this effect was seen with both Ach, which stimulates Muscarinic/Ach receptors and KCl, which depolarizes the plasma membrane to induce Ca^2+^ influx^11^,^12^, we concluded that this effect was not due to abnormal receptor function. Furthermore, pretreatment with recombinant BPIFA1 protein at a physiologically relevant dose^13^ (10 μM) for 1 h suppressed contraction (Fig. 1f), suggesting that BPIFA1 is an EDSMRF.

### BPIFA1 regulates ASM contraction *in vitro*

**Figure 2:**
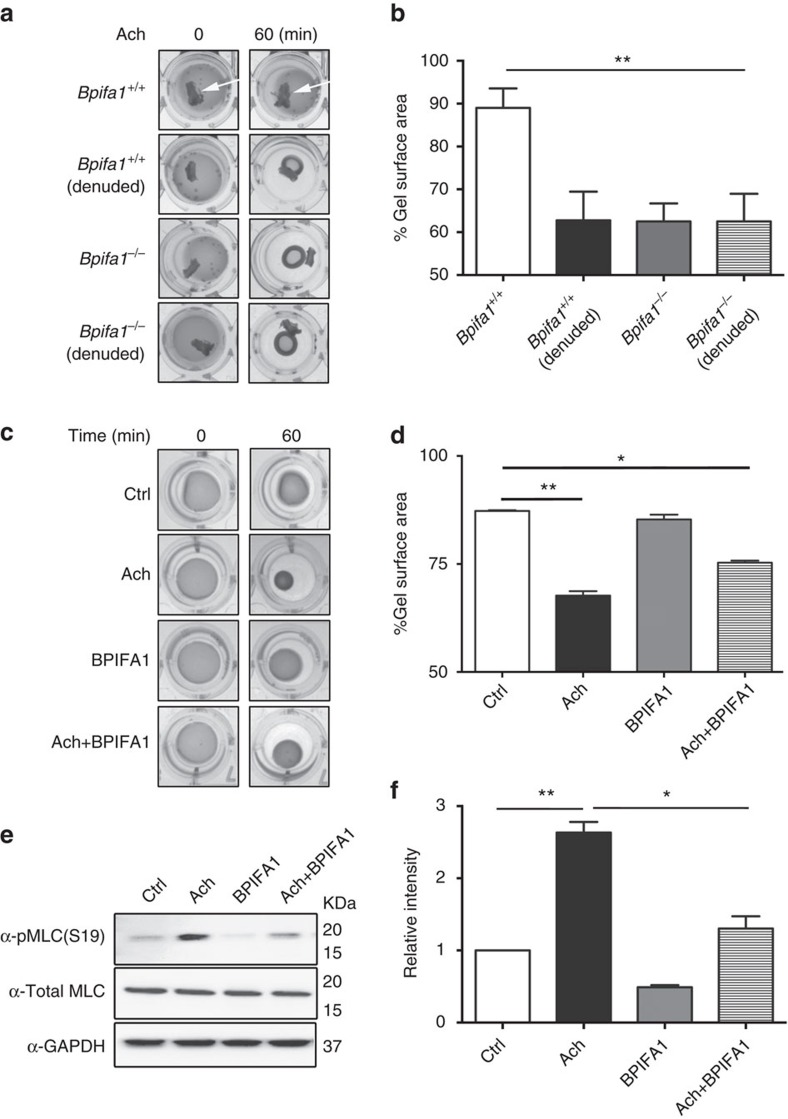
BPIFA1 decreases airway smooth muscle (ASM) contractility by suppressing myosin light chain (MLC) phosphorylation. Human ASMCs were grown in type I collagen matrices in 24-well plates. Gel contractions were measured as indicated over 60 min. (**a**) Human ASMCs were co-cultured with whole and denuded tracheal rings from *Bpifa1*^*+/+*^ or *Bpifa1*^*−*/*−*^ mice for 48 h before contraction was induced with Ach. White arrows show the location of excised tracheas on the cultures. (**c**) ASMCs were pretreated with BPIFA1 (10 μM) or vehicle for 1 h and gel contraction was then measured±Ach (100 μM). Representative images of gel contraction are shown. (**b**,**d**) Summary of data from **a** and **c**, respectively, expressed as the % decrease in gel surface area at 60 min (all *n*=3 per group). (**e**) Representative immunoblots probed for total and phosphorylated MLC. (**f**) Mean densitometry taken from **e** (*n*=3). Data in **b**,**d** and **f** are mean±s.e.m. The data were analysed using one-way ANOVA followed by Tukey *post hoc* analysis. *P<0.05, **P<0.01.

Due to the potential importance of this observation, we further investigated the ASM–BPIFA1 interaction in ASM cells (ASMCs) cultured in collagen matrices. We observed that ASMC contraction was significantly reduced in the presence of *Bpifa1*^*+/+*^ tracheas with intact epithelium. In contrast, denuded tracheas from both genotypes and whole *Bpifa1*^*−*/*−*^ tracheas showed no inhibitory effect on ASMC contraction (Fig. 2a,b). Similarly, pre-incubation with recombinant BPIFA1 for 1 h reduced ASMC contraction (Fig. 2c,d), suggesting that epithelium-derived BPIFA1 is responsible for modulating ASM contractility. In ASMC, contraction is regulated by cross-bridge formation between phosphorylated myosin light chain (MLC) and actin. Ach enhances MLC phosphorylation in a Ca^2+^-dependent manner and therefore enhances contraction^14^. MLC phosphorylation was increased by Ach, whereas pre-treatment with BPIFA1 decreased both basal and induced MLC phosphorylation (Fig. 2e,f and Supplementary Fig. 2).

### BPIFA1 levels are reduced in asthma-derived HBECs

**Figure 3:**
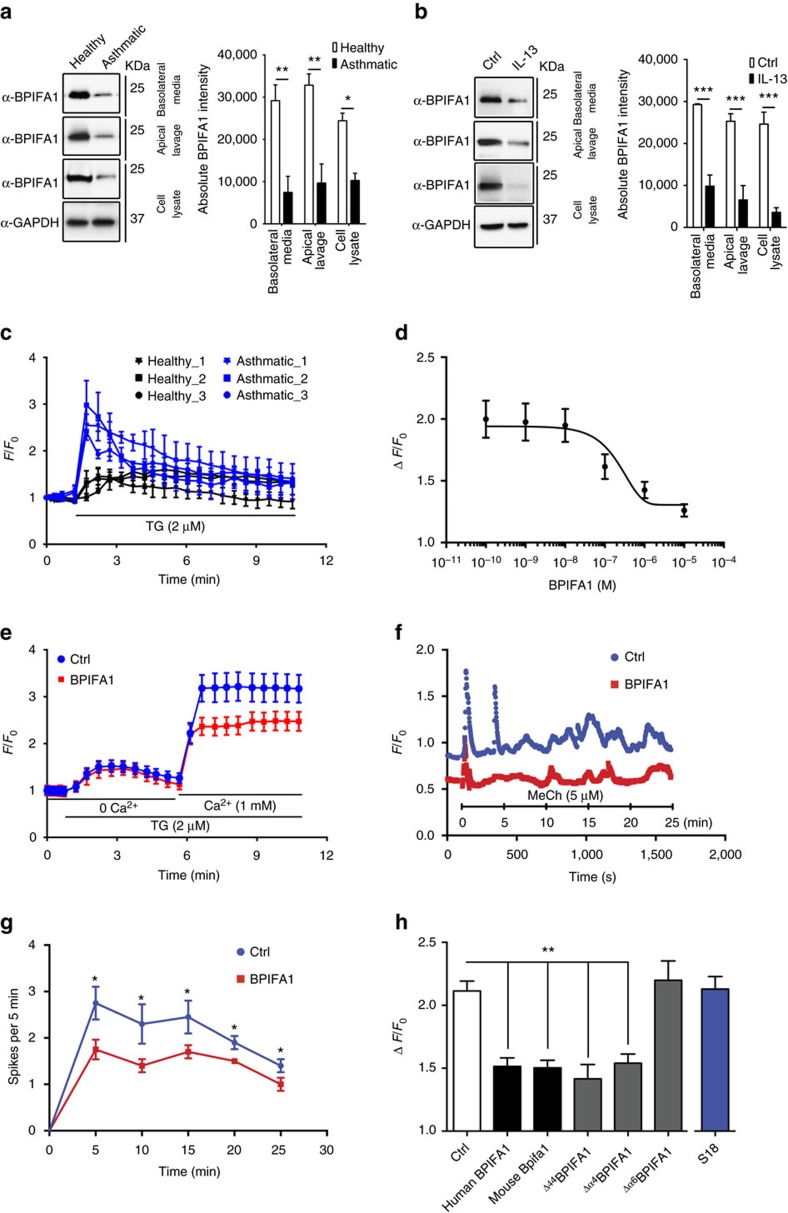
BPIFA1 from normal but not asthmatic HBECs is secreted basolaterally and blocks Ca^2+^ influx in ASMCs. (**a**) Representative immunoblots (left panel) and densitometry (right panel) showing BPIFA1 from healthy and asthmatic HBECs (*n*=6 per group). (**b**) Representative immunoblots (left panel) and densitometry (right panel) showing BPIFA1 levels in vehicle and IL-13 exposed HBECs (*n*=3 per group). (**c**) Mean changes in fura-2 fluorescence in ASMC with time, as an indicator of cytosolic Ca^2+^. Serosal media from normal and asthmatic HBECs were co-incubated with human ASMCs for 1 h. (**d**) BPIFA1 inhibits TG-induced cytosolic Ca^2+^ increases in a dose-dependent manner. ASMCs were incubated with BPIFA1 and changes in the fura-2 emission ratio over time were recorded. Δ*F*/*F*_0_ represents the average peak fluorescent intensity changes of three independent experiments. (**e**) Mean change in the fura-2 emission ratio over time following TG and the addition of extracellular Ca^2+^. (**f**) Typical Ca^2+^ oscillations in ASMCs±BPIFA1 in response to 5 μM Mch. (**g**) Summary of Ca^2+^ oscillation frequency data following 5 μM Mch. (**h**) Summary of peak fluorescence ratio changes when ASMCs were pre-incubated with BPIFA1 mutants or peptides (*n*=3 per group). Data in **a**–**e**,**g** and **h** are mean±s.e.m. The data were analysed using Student's *t*-test in **a** and **b**, Mann-Whitney test in **g** and one-way ANOVA followed by Tukey *post hoc* analysis in **h**. *P<0.05, **P<0.01 and ***P<0.0001 different to control.

We next measured BPIFA1 protein levels in primary HBECs derived from healthy and asthmatic donors. Consistent with our data from the sputum samples (Fig. 1a,b), immunoblot analyses revealed significantly decreased levels of BPIFA1 in lysates, basolateral media and apical lavage of HBECs derived from asthma patients compared with non-asthmatic controls (Fig. 3a and Supplementary Table 2). Normal HBECs exposed to the asthma-associated T-helper 2 cell (Th2) cytokine IL-13 showed similar decreases in BPIFA1 levels (Fig. 3b). Consistent with the previous studies^10^, we also detected a decrease in *BPIFA1* mRNA after IL-13 exposure (Supplementary Fig. 3). On the basis of these data, we hypothesize that diminution of BPIFA1 in asthmatic tissue may be closely associated with the Th-2 response. Of note, exposure to the β2-adrenergic agonist albuterol enhanced BPIFA1 secretion in both basal media and apical lavage of normal HBECs without affecting overall *BPIFA1* mRNA and protein levels (Supplementary Fig. 4).

### BPIFA1 inhibits store-operated Ca^2+^ entry in ASM

Since BPIFA1 downregulated MLC phosphorylation, we next investigated whether BPIFA1 regulated ASMC Ca^2+^ signalling. When ASMCs were exposed to serosal media from healthy HBECs, or to recombinant BPIFA1, the thapsigargin (TG)-induced Ca^2+^ increase was significantly reduced in a dose-dependent manner (Fig. 3c,d and Supplementary Fig. 5a–c). After TG exposure, Ca^2+^ enters the cytoplasm from multiple sources including the endoplasmic reticulum (ER) and the extracellular milieu^15^. In the absence of extracellular Ca^2+^, TG triggered ER Ca^2+^ release and only moderately raised cytoplasmic Ca^2+^, whereas reintroduction of Ca^2+^ extracellular Ca^2+^ induced SOCE and further elevated cytosolic Ca^2+^ (Fig. 3e). Pre-treatment with BPIFA1 had no effect on ER Ca^2+^ release, but significantly suppressed SOCE (Fig. 3e and Supplementary Fig. 5d). Since SOCE supports intracellular Ca^2+^ oscillations^16^, we also examined the role of BPIFA1 in regulating this phenomenon. Pre-treatment with BPIFA1 for 1 h significantly attenuated Mch-induced Ca^2+^ oscillations (Fig. 3f,g), indicating that BPIFA1 inhibits the Ca^2+^ entry required for oscillations.

To map out the structural region within BPIFA1 that was responsible for these effects, we used a series of BPIFA1 mutants/peptides and tested their ability to suppress Ca^2+^ signalling. Inhibition was not different for mouse and human BPIFA1 (Fig. 3h). BPIFA1 utilizes its N-terminal ‘S18' region to regulate epithelial Na^+^ channel (ENaC) plasma membrane density^17^,^18^. The S18 peptide^17^ did not inhibit SOCE, suggesting that the action of BPIFA1 was ENaC-independent. BPIFA1 lacking its N-terminal S18 region (^Δ44^BPIFA1) or deletion of alpha helix 4 (^Δα4^BPIFA1) still inhibited Ca^2+^ signalling. However, deletion of alpha helix 6 (^Δα6^BPIFA1) significantly abolished its ability to inhibit Ca^2+^ signalling (Fig. 3h), indicating that the C-terminal α6 helix is required to inhibit SOCE.

### BPIFA1 binds to and internalizes Orai1

**Figure 4:**
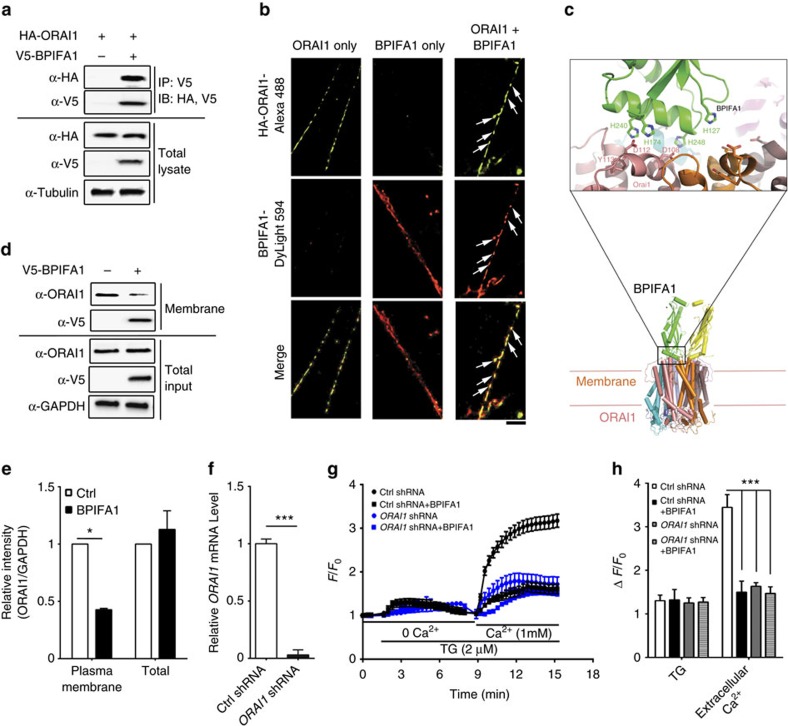
BPIFA1 modulates SOCE by interacting with Orai1. (**a**) Immunoprecipitation analysis was performed using cell lysates from HEK293T cells cotransfected with V5- BPIFA1 and HA-Orai1. HA-Orai1 was pulled down by V5-BPIFA1. Data represent three independent immunoprecipitations. (**b**) Ground-state depletion super-resolution images of HA-tagged Orai1 (Green) and DyLight 594 labelled BPIFA1 (Red) in ASMCs. Scale bar is 2.5 μm. (**c**) BPIFA1–ORAI1 complex model: a model of human Orai1 was created using the structure of *Drosophila* Orai1 as a guide (RCSB 4HKR), and the structure of human BPIFA1 (RCSB 4KGH) was manually docked onto the Orai1 hexamer by aligning conserved electrostatic features. (**d**) Human ASMCs were incubated with 10 μM BPIFA1 for 4 h, followed by surface biotinylation and immunoblot using indicated antibodies. (**e**) Mean densitometry of plasma membrane and total Orai1/GAPDH and expressed as relative intensity (*n*=3). (**f**) Human ASMCs were transfected with control and *ORAI1* shRNA respectively. mRNA levels of Orai1 were measured by qRT–PCR. (**g**) Average traces of Ca^2+^ imaging using fura-2. Human ASMCs were transfected with control and *ORAI1* shRNA respectively, for 72 h. After incubating with or without 10 μM BPIFA1 for 1 h, fura-2 emission ratio was then recorded over time. (**h**) Summary of peak fluorescent ratio change in the presence of TG (*n*=3 per group). Data in **e**–**h** are mean±s.e.m. The data were analysed using Mann-Whitney in **e** and **f**, one-way ANOVA followed by Tukey *post hoc* analysis in **h**. *P<0.05, ***P<0.001 different to control.

Since Orai1 mediates SOCE and both are hyperactive in murine asthma models where BPIFA1 is diminished^5^,^19^, we tested whether these proteins interact. BPIFA1 and Orai1, but not an alternate Ca^2+^ channel (transient receptor potential cation channel 3, TRPC3), could be co-immunoprecipitated (Fig. 4a and Supplementary Fig. 6). Using ground state depletion (GSD) super resolution microscopy to give a resolution of 30 nm^2^ per pixel, BPIFA1 and Orai1 were found to co-localize in ASMC plasma membranes after 1 h of co-incubation (Fig. 4b). The structures of Orai1 and BPIFA1 have been solved^9^,^20^ and our docking model suggested that the histidines in BPIFA1's α6 helix, fit into the highly conserved negatively charged regions of Orai1 (Fig. 4c). Surface biotinylation/immunoblot and confocal microscopy demonstrated that plasma membrane Orai1 levels decreased by ∼50% after 4 h of BPIFA1 exposure (Fig. 4d,e). To verify that Orai1 was BPIFA1's target, we knocked down endogenous *ORAI1* in ASMCs by shRNA, as confirmed by both qPCR (Fig. 4f) and immunoblot (Supplementary Figs 7a and 8a). *ORAI1* knockdown decreased BPIFA1 binding to ASMC plasma membranes (Supplementary Fig. 7) and the ability of BPIFA1 to inhibit Ca^2+^ influx and ASMC contrition *in vitro* were also lost (Fig. 4g,h, Supplementary Figs 9 and 10). Additionally, phosphorylation of myosin light chain phosphatase (MYPT1) at threonine 583, which was reportedly induced by Ach in mice tracheas^21^, was downregulated by BPIFA1 (Supplementary Fig. 8). Taken together, our data suggest that BPIFA1 binds to and inhibits Orai1 to block Ca^2+^ influx in ASMC, resulting in a decrease in MLC phosphorylation and ASM contractility.

## Discussion

Our previous studies, and those conducted elsewhere, have focused on BPIFA1's secretion into the lung lumen^18^,^22^,^23^. For example, BPIFA1 negatively regulates Na^+^ absorption across airway epithelia by binding extracellularly to β-ENaC^18^,^24^. We note that BPIFA1's S18 region, which inhibits ENaC, is located N-terminally^12^, while the α6 helix, which antagonizes Orai1, lies close to BPIFA1's C terminus. Thus, these domains are in distinct regions of BPIFA1, which may support their differing functions. Chu and colleagues demonstrated that *Bpifa1*^*−*/*−*^ mice have a more severe phenotype than WT mice when sensitized by ovalbumin, including eosinophilic inflammation^25^. These authors hypothesized that apically secreted BPIFA1 normally ‘mopped up' excess bacterial lipopolysaccharide and that after allergen exposure, reduced BPIFA1 levels increased lipopolysaccharide, leading to an exaggerated allergen response. However, they did not factor basolaterally secreted BPIFA1 into their model. It is likely therefore, that the ovalbumin-induced decrease in BPIFA1 levels also contributed to AHR via the dysregulation of BPIFA1–Orai1 interactions. We found that BPIFA1 levels were significantly diminished in sputum derived from asthma patients (Fig. 1a,b) and that a similar decrease in BPIFA1 levels was observed in apical secretions from both asthmatic and IL-13-exposed HBECs (Fig. 3a,b). Perhaps surprisingly, we demonstrated that BPIFA1 was also basolaterally secreted from airway epithelia, and that basolateral BPIFA1 secretions were greatly diminished in asthmatic and IL-13 exposed HBECs (Fig. 2a,b). Mirroring these decreases, we found that asthma and IL-13 downregulated *BPIFA1* mRNA and intracellular protein levels (Fig. 2a,b and Supplementary Fig 3). Why asthmatic HBECs continued to display reduced BPIFA1 levels remains to be determined. They may have a ‘memory' of the host inflammation and/or exhibit as yet unknown genetic variations that predispose them to reduced BPIFA1 levels.

BPIFA1 binds extracellularly to Orai1, leading to Orai1's internalization, inhibition of SOCE and a decrease in ASM contraction (Figs 2 and 4), a process that is deranged in asthmatic airways (Figs 1 and 3). Consistent with these data, we have also discovered a hitherto unnoticed phenotype of the *Bpifa1*^*−*/*−*^ mice, that is, they exhibit spontaneous AHR that is reversed in excised tracheas by the addition of recombinant BPIFA1 (Fig. 1c–f). The inhibitory effect of BPIFA1 on ASM contraction also requires an intact of airway epithelia. Indeed, denuding tracheas from *Bpifa1*^*+/+*^ mice abolished their ability to suppress ASM contraction *in vitro*, an effect that was absent from whole or denuded *Bpifa1*^*−*/*−*^ tracheas (Fig. 2a,b). Taken together, these data strongly suggest that BPIFA1 is indeed an EDSMRF. BPIFA1 is expressed in epithelia of the trachea and bronchi, but not the alveoli^26^,^27^, suggesting that is able to function as an EDSMRF in the conducting airways. However, whether other EDSMRFs exist remains to be determined.

We focused on basolaterally secreted BPIFA1's ability to regulate ASM. However, the lack of apically secreted BPIFA1 may also have consequences for airway epithelia. Indeed, mucus is dehydrated in asthmatic airways^28^ and since a lack of BPIFA1 is also predicted to increase ENaC activity, this may indicate that mucus dehydration in asthma patients is in part driven by BPIFA1 deficiency and abnormal ENaC-led transepithelial Na^+^ and water absorption. In addition, since mucus secretion from goblet cells is Ca^2+^-dependent^29^, increased SOCE in the absence of BPIFA1 may also contribute to the mucus hypersecretion phenotype seen in asthma patients. For example, our data predict that in BPIFA1's absence, the purinergic stimulation of goblet cells would result in a greater cytosolic Ca^2+^ response and increased mucin secretion. BPIFA1 notwithstanding, Th2-driven goblet cell metaplasia helps drive the mucus hypersecretion component^30^. The transcription factors SAM-pointed domain-containing ETS-like factor (SPDEF) and forkhead ortholog A3 (FOXA3) are abnormally regulated in asthma leading to goblet cell metaplasia^31^,^32^. Whether SPDEF/FOXA3 activity is involved in reducing BPIFA1 expression in asthmatic patients remains to be determined. β2-agonists, which are a common asthma treatment, can increase BPIFA1 expression^33^, whereas our data suggest that the short-term β2-agonist albuterol only enhances BPIFA1 secretion (Supplementary Fig. 4). Therefore, a better understanding of how BPIFA1 expression is modulated by different categories of β-agonists and other mainstream therapies including glucocorticoids in asthma-derived airway epithelia is needed to develop novel, targeted therapies for treating asthma.

Although our study focused on the diminished expression of BPIFA1 and its target Orai1 in ASM, it is worth noting that other research groups have demonstrated the dysregulation of other plasma membrane proteins. For instance, calcium-sensing receptor (CaSR) is upregulated in asthmatic ASM leading to AHR and inflammation^34^. CD38 is also elevated in asthmatic ASM and influences Ca^2+^ homeostasis/ASM contractility^35^. It is unclear whether BPIFA1 interacts with these proteins. However, replacing BPIFA1 may normalize Ca^2+^ homeostasis and reduce AHR irrespective of CaSR and CD38 activity. Perhaps surprisingly, we found that BPIFA1 also abrogated KCl-induced increases in cytosolic Ca^2+^ and ASM contractions (Fig. 1 and Supplementary Fig. 9). We also observed that Orai1 is involved in the KCl response and indeed, we find that knockdown of Orai1 reduces KCl-induced increases in cytosolic Ca^2+^ (Supplementary Fig. 9). While these data are unconventional, it has been previously been demonstrated that elevations in extracellular KCl trigger ATP release^36^,^37^. This is not entirely surprising since many factors (including but not limited to mechanical and osmotic stress also induce ATP release). Furthermore, the ATP release channel PANX1 is also stimulated by extracellular K^+^ (ref. 38). Thus, while further experimentation will be required, we propose that KCl-induced ATP release stimulates purinergic receptors to actives SOCE/Orai1.

Beyond asthma, altered BPIFA1 expression is associated with several types of cancer including nasopharyngeal carcinoma^39^ and salivary gland cancers^40^. Changes in Orai1 are also associated with increased cancer metastasis^41^. Thus, we speculate that altered BPIFA1 expression may contribute to abnormal Orai1-dependent cell growth, although further mechanistic studies will be required to confirm or refute this link. In conclusion, our findings that BPIFA1 is secreted basolaterally, where it acts as an EDSMRF to modulate SOCE and ASM contraction, are fundamentally novel advances in understanding asthma pathogenesis that provide a direct link between epithelial dysfunction and AHR, and for future treatments for asthma.

## Methods

### Human sputum sample collection

All studies were approved by the UNC Institutional Review Board and informed consent was obtained from all subjects. Demographic information is included in Supplementary Table 1. Sputum induction was performed as previously published^42^,^43^, either by spontaneous expectoration or via sputum induction for different protocols. Spontaneous expectoration was performed as follows: subjects were informed not to deliver saliva or nasal secretions into the specimen cup labelled ‘sample', but rather if present, they were expectorated into the specimen cup labelled ‘waste'. Before any attempt to deliver a spontaneous sputum sample, all subjects underwent a three-step cleansing routine as follows: (1) rinse the mouth and gently gargle with water and expectorate the contents into the ‘waste' cup; (2) scrape and clear the back of the throat and expectorate the contents into the ‘waste' cup; (3) blow the nose. Following these three cleansing steps, coughing attempts from the chest (and not the throat) were attempted. Subjects were instructed not to scrape the back of the throat while coughing up sputum from the chest. All sputum specimens were delivered into the specimen cup marked ‘sample'. The cough attempts were repeated until no further sputum was being expectorated from the chest. The spontaneous sputum sample was kept on ice (4 °C) during the entire spontaneous sputum procedure. The induced sputum procedure was administered as follows: following the acquisition of a baseline FEV1, a 10 and 20% fall from this baseline FEV1 value was calculated and recorded. Subjects then were administered successive increasing concentrations of saline (3, 4 and 5%) using a Devilbiss UltraNeb 99 ultrasonic nebulizer (Sunrise Medical) where the nebulizer flow rate and aerosol generation rate were adjusted to each subject's tolerance level to allow inhalation without discomfort. Each saline inhalation period lasted 7 min and subjects inhaled saline at tidal volume. Subjects were instructed to not swallow saliva or saline during the inhalation periods but rather expectorate it into the ‘waste' cup if there was a need. At the end of each 7 min inhalation period, subjects underwent the three-step cleansing procedure followed by chesty (not throat) cough attempts as described above for spontaneous sputum expectoration. When no further sample could be expectorated, as typically indicated by a dry sounding cough, FEV1 was assessed. If the fall in FEV1 was <10% from the pre-procedure baseline FEV1, the next highest dose of saline was administered and the next 7 min inhalation period commenced. If the fall in FEV1 was >10% but <20%, the same saline concentration was administered for the next 7 min inhalation period. If the fall in FEV1 was 20% or greater, the sputum induction procedure was terminated and not restarted and the patient was given rescue therapy as needed. The collected sputum sample after each 7 min inhalation period was pooled into one specimen cup and kept on ice (4 °C) during the entire induction procedure. All three saline inhalation periods were performed unless the FEV1 fell by 20% or greater, or a subject wished to terminate the procedure early. All subjects' FEV1 had to return to within 5% of baseline before discharge.

### Animals and measurement of airway resistance

Animals were cared for according to the guidelines, and all procedures were approved by UNC Animal Care and Use Committee. *Bpifa1*^*−*/*−*^ knockout and *Bpifa1*^*+/+*^ littermate control mice were kind gifts from Dr Paul B. McCray Jr at University of Iowa, and were bred and housed in a vivarium at The University of North Carolina at Chapel Hill. Airway resistance was measured in anaesthetized 8-week-old male mice similar to previously described^44^. Basal resistance measurements were made every 10 s for 1 min before serially challenging mice with aerosolized methacholine (Mch) at the following concentrations: 10 mg and 20 mg ml^−1^. Mice were administered each concentration of Mch for 10 s before recording, using a flexiVent (SCIREQ) at 12.5 s intervals for 2.5 min immediately following each challenge period.

### DNA constructs

Yellow fluorescent protein (YFP)-tagged human *ORAI1* and Myc-tagged TRPC3 were gifts from Dr Craig Montell (Addgene plasmid # 25902) and Dr Anjana Rao (Addgene plasmid # 19756), respectively. HA-tagged human *ORAI1* was a generous gift from Dr Patrick G. Hogan at La Jolla Institute for Allergy & Immunology. pcDNA3.1(+)-V5-BPIFA1 was generously provided by Dr Colin D. Bingle at the University of Sheffield.

### Cell culture and transfection

Human ASMCs were generous gift from Dr Raymond B. Penn at Thomas Jefferson University. Rat ASMCs were kindly provided by Dr Mohamed Trebak at Pennsylvania State University. ASMCs were maintained in Dulbecco's Modified Eagle Medium: Nutrient Mixture F-12 (DMEM/F12; Thermo Fisher) supplemented with 10% fetal bovine serum (FBS; Sigma Aldrich) and 0.1% penicillin–streptomycin (Thermo Fisher). HEK293T cells (catalogue number: CRL-3216) were purchased from ATCC and maintained in DMEM (Thermo Fisher) in the presence of 10% FBS and 0.1% penicillin–streptomycin. HBECs were obtained from freshly excised bronchial specimens derived from normal and asthmatic subjects and were harvested as previously described under protocol #03-1396 approved by the University of North Carolina at Chapel Hill Biomedical Institutional Review Board^45^. Informed consent was obtained from all donors or authorized representatives of the donors. Briefly, HBECs were extracted from excess surgical pathology or autopsy specimens procured through cooperating surgeons and pathologists using protocols in accordance with relevant regulations. Airways were dissected by removing all excess connective tissue and cutting into 5–10 cm segments. Specimens were dissociated over 4–24 h, depending on the size of the tissue, in a 15 ml tube containing 9 ml tissue wash medium (Joklik Minimum Essential Medium <JMEM> supplemented gentamicin <50 μg ml^−1^> and amphotericin <0.25 μg ml^−1^>) plus 1 ml protease solution (1% Protease XIV with 0.01% DNase <10 × stock>, Sigma Aldrich). HBECs were then cultured at an air–liquid interface in a modified bronchial epithelial growth medium with 5% CO_2_ at 37 °C and were used 3–4 weeks after the seeding on 12 mm T-clear inserts (Corning). Demographic information for healthy and asthmatic donors is included in Supplementary Table 2. Short hairpin RNA (shRNA) plasmid against *ORAI1* and scrambled control shRNA plasmid were purchased from Sigma Aldrich. Transfections of all plasmid DNA and shRNA were performed using Lipofectamine 2000 reagent (Thermo Fisher) according to the manufacturer's instructions.

### Immunoprecipitation and immunoblot analysis

Rabbit anti-Orai1 (1:1,000), anti-HA epitope (1:2,000) and anti-myc epitope (1:1,000) (Santa Cruz), anti-GFP (1:1,000), anti-phospho-myosin light chain (S19) (1:1,000), anti-total myosin light chain (1:1,000), anti-phospho-myosin light chain phosphatase (T853) (1:1,000), anti-phospho-myosin light chain phosphatase (T696) (1:1,000), anti-total myosin light chain (1:1,000), anti-total-myosin light chain phosphatase (1:1,000), anti-GAPDH (glyceraldehyde-3-phosphate dehydrogenase, 1:3,000) (Cell Signaling Technology); mouse anti-V5 epitope (1:2,000, Thermo Fisher); goat anti-BPIFA1 (1:2,000, R&D Systems); peroxidase-conjugated donkey anti-mouse IgG (H+L), peroxidase-conjugated donkey anti-rabbit IgG (H+L), peroxidase-conjugated donkey anti-goat IgG (H+L) (all 1:3000, Jackson ImmunoResearch) were purchased from commercial sources. To detect protein expression in total cell lysates, cells were lysed in Pierce IP lysis buffer with 25 mM Tris-HCl pH 7.4, 150 mM NaCl, 1% NP-40, 1 mM EDTA, 5% glycerol, supplemented with 1 × proteinase inhibitor cocktail (PIC) (Roche), followed by sodium dodecyl sulfate (SDS)–polyacrylamide electrophoresis and immunoblot. For immunoprecipitation, cell lysates were collected at 48 h post transfection in Pierce IP lysis buffer in the presence of 1 × PIC. Cell lysates were pre-cleared with protein A/G agarose beads and then incubated 1 μg of antibodies against HA or V5 with protein A/G agarose beads on a rotator at 4 °C overnight. After three washes with IP lysis buffer, immunoprecipitated complexes were eluted in sample buffer (50 mM Tris-HCl (pH 6.8), 2% SDS, 10% glycerol, 5% (v/v) β-mercaptoethanol (BME), 0.1% bromophenol blue) by heating the samples at 95 °C for 5 min, electrophoresed through SDS–polyacrylamide gels, and subjected to immunoblot analysis. All the uncropped scans are provided in Supplementary Fig. 11.

### Calcium imaging

Calcium imaging using fura-2 was adapted from a previously published protocol with minor modifications^46^. Briefly, ASMCs were loaded with 2 μM fura-2-AM (Thermo Fisher) and 24 h old serosal media from HBECs or media containing recombinant BPIFA1 at 37 °C for 1 h. ASMCs were then washed with a standard Ringer's solution (101 mM NaCl, 12 mM NaHCO_3_, 1.2 mM MgCl_2_, 1.2 mM CaCl_2_, 0.2 mM KCl, 24 mM HEPES, 10 mM glucose, pH 7.4) or with Ca^2+^-free Ringer's solution as indicated. Cultures were then placed in the Ringer's solution, and images were collected with a 40 × 1.4 NA Plan Fluor oil objective on a Nikon Ti-S inverted microscope. Fura-2 fluorescence was acquired alternately at 340 and 380 nm (emission >450 nm) using LUDL filter wheels, obtained with an Orca FLASH 4.0 CMOS camera (Hammamatsu) and controlled with HCImageLive software (Hammamatsu). Cell bodies were identified as individual regions of interest (ROIs). Background subtraction was performed using a cell-free region as the background region. Signals were converted to relative changes (*F*/*F*_0_) where *F*_0_ was the ratio of the average fluorescent intensity (340/380) of ROIs at 0 time point. Typically, 20 cells per coverslip were recorded. Δ*F*/*F*_0_ represents average peak fluorescent intensity changes of three independent experiments.

### Myography of murine tracheal rings

Tracheas were excised from 8 weeks old male *Bpifa1*^*+/+*^ and *Bpifa1*^*−*/*−*^ mice. After removing excessive connective tissue, the trachea was cut into ∼4 mm rings, mounted on a DMT 620M myography apparatus (DMT) and allowed to rest in modified Ringer's solution (119 mM NaCl, 4.7 mM KCl, 1.17 mM MgSO_4_, 1.18 mM KH_2_PO_4_, 2.5 mM CaCl_2_, 25 mM NaHCO_3_, 0.027 mM EDTA, 5.5 mM glucose) with continuous oxygenation (95% O_2_/5% CO_2_) at 37 °C for 20 min with no applied tension. Optimal passive tension (1 mN) was then applied to the rings. After stable tension was achieved, the baseline force was then recorded. The induced contractile force of each ring was assessed by measuring contraction stimulated by 60 mM KCl or 100 mM Ach (unless indicated otherwise), respectively. Rings were bilaterally exposed to recombinant BPIFA1 or vehicle control for 1 h before Ach/KCl addition.

### Cell contraction assay

The cell contraction assay was performed using a standard commercially available kit (Cell Biolabs). Human ASMCs were harvested and re-suspended in DMEM, two parts of cells were mixed with eight parts of collagen gel lattice mixture and plated for 1 h at 37 °C. After the gel solidified, 1 ml of medium was added and incubated for 48 h. Next, the gels were released from the sides of wells, and the images were taken using a ChemiDoc MP imager (Bio-Rad) at 0 and 1 h after adding indicated reagents. The changes of collagen gel surface areas were analysed using ImageJ software and normalized to the gel's area at *t*=0 min.

### Protein purification and fluorescent labelling

cDNA of full length and truncated BPIFA1 was transformed in BL21-Codon Plus competent cells (Agilent Technologies), and purified as previously described^24^. S18 peptide was synthesized and purified by the UNC Microprotein Sequencing and Peptide Synthesis Facility, as described previously^17^. Fluorescent labelling was done using DyLight 594 or DyLight 633 NHS ester (Thermo Fisher) by following the manufacturer's instructions.

### Surface labelling and super resolution microscopy

Human ASMCs were grown on 22 mm^2^ glass coverslips and transfected with HA-*ORAI1* DNA. Twenty-four hours after transfection, cells were fixed with 4% paraformaldehyde (PFA) in PBS, followed by surface labelling with mouse anti-HA antibody (BioLegend) and Alexa 488 goat anti-mouse antibody (Thermo Fisher) at 4 °C. After washing 5 × with ice-cold PBS, cells were incubated with 5 μM BPIFA1-DyLight 594 for 1 h at 4 °C before imaging. Before mounting, 90 μl β-Mercaptoethylamine (MEA) was added to the cavity of a depression slide to deplete oxygen. Coverslips were sealed with twinsil (Picodent). Super-resolution images were captured with a commercial Leica GSD super-resolution microscope using a 160 × 1.43 NA objective. GSD was performed by exciting the fluorophore with 488 and 642 nm solid-state lasers. In some cases, to facilitate exit from the ground state, back pumping was applied using the 405 nm laser as per the manufacturer's instruction.

### Fluorescent BPIFA1 binding assay

Human ASMCs were transfected with scrambled (control) shRNA or *ORAI1* shRNA, respectively. Seventy-two hours post transfection, cells were treated with or without BPIFA1-DyLight 633 for 1 h, then washed 5 × with ice-cold Ringer's solution. ASMC-bounded BPIFA1-Dylight 633 was detected using a Leica TCS SP8 63 × 1.4 NA oil immersion objective using the Leica Application Suite X software (Leica). In addition, fluorescent intensity of ASMC-bounded BPIFA1-Dylight 633 was detected by a fluorescent plate reader (Infinite M1000, Tecan). Cells were also stained with calcein AM (Thermo Fisher) as a cell number control. Relative fluorescent intensity was calculated by normalizing fluorescent intensity at 658–526 nm.

### Surface biotinylation

Human ASMCs were treated with or without BPIFA1 for 4 h. Cells were then washed with prechilled PBS^++^ (phosphate-buffered saline supplemented with 1 mM CaCl_2_ and 1 mM MgCl_2_), and then labelled with 0.5 mg ml^−1^ sulfo-NHS-biotin (Thermo Fisher) in borate buffer (85 mM NaCl, 4 mM KCl, 15 mM Na_2_B_4_O_7_, pH 9.0) while tumbling gently for 30 min on ice. ASMCs were incubated in PBS^++^ buffer supplemented with 10% FBS for 20 min at 4 °C to quench free biotin. Cells were washed again three times with chilled PBS^++^ and proteins were extracted as previously described using lysis buffer (0.4% sodium deoxycholate, 1% NP-40, 50 mM EGTA, 10 mM Tris-Cl, pH 7.4) supplemented with 1 × PIC. Total inputs were taken from whole-cell samples representing 4% of total protein. Solubilized proteins were incubated with 100 μl of neutravidin beads (Thermo Fisher) overnight while rotating at 4 °C. Samples were washed three times with lysis buffer. Bead-bound proteins were then eluted and subjected to immunoblot analysis.

### Statistical analysis

All data are presented as the mean±s.e.m. for *n* experiments. Differences between means were tested for statistical significance using paired or unpaired *t*-tests or their non-parametric equivalent as appropriate to the experiment. Differences between groups were judged using analysis of variance. From such comparisons, differences yielding *P*≤0.05 were judged to be significant. GraphPad Prism software was used for statistical analysis.

### Data availability

All raw and analysed data are available from the authors upon request.

## Additional information

**How to cite this article**: Wu, T. *et al*. Identification of BPIFA1/SPLUNC1 as an epithelium-derived smooth muscle relaxing factor. *Nat. Commun.* **8**, 14118 doi: 10.1038/ncomms14118 (2017).

**Publisher's note**: Springer Nature remains neutral with regard to jurisdictional claims in published maps and institutional affiliations.

## Supplementary Material

Supplementary Information Supplementary figures 1-11 and supplementary tables 1-2.
