# Supplementary material for: Identification of BPIFA1/SPLUNC1 as an epithelium-derived smooth muscle relaxing factor
Source: Nat Commun. 2017 Feb 6;8:14118. doi: 10.1038/ncomms14118 (PMC5303822; doi:10.1038/ncomms14118)
Supplement: Supplementary Information — Supplementary figures 1-11 and supplementary tables 1-2. [file ncomms14118-s1.pdf]

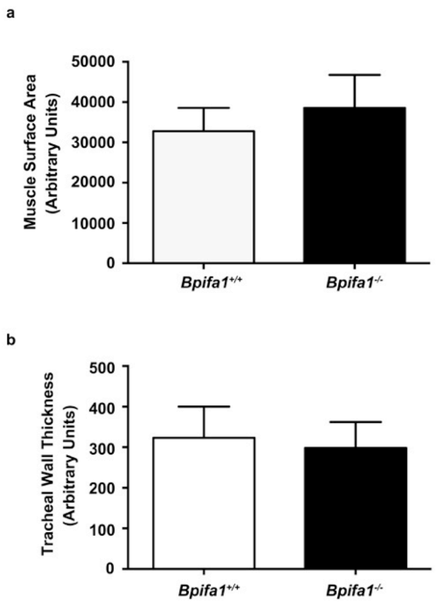


**Supplementary Figure 1. *Bpifa1$^{-/-}$* murine tracheal smooth muscles show no sign of hypertrophy.** Quantification of smooth muscle from *Bpifa1$^{+/+}$* and *Bpifa1$^{-/-}$* mice tracheas was expressed as average surface areas **(a)** and tracheal wall thickness **(b)** in arbitrary units**.** Images were analyzed using ImageJ software. Two sections/mouse, 3 mice/genotype were analyzed. Data are mean±s.e.m and were analyzed using student's *t*-test.

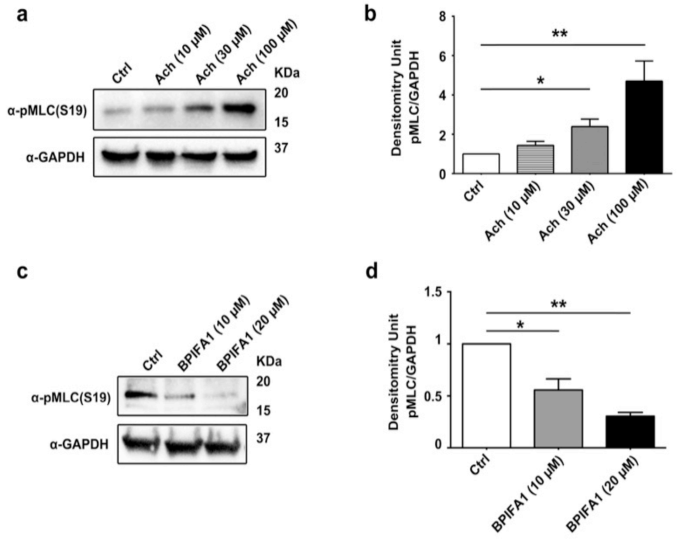


**Supplementary Figure 2. BPIFA1 decreases MLC phosphorylation in a dose-dependent fashion.** Immunoblots were probed with an antibody that recognizes phosphorylated MLC and normalized to GAPDH. **(a)** Representative immunoblot demonstrating that Ach increases MLC phosphorylation. **(b)** Intensity of immunoblots from (a) were quantified using Image J, normalized to GAPDH and expressed as relative intensity (n=3). (c) Representative immunoblot demonstrating that BPIFA1 increases MLC phosphorylation. **(d)** Bar graph of immunoblot intensity from **(c)** were quantified using Image J, normalized to GAPDH and expressed as relative intensity (n=3). Data in b and d are mean±s.e.m. The data were analyzed using one-way ANOVA followed by Turkey post hoc analysis. * and ** indicate P<0.01and P<0.05 different to control respectively.

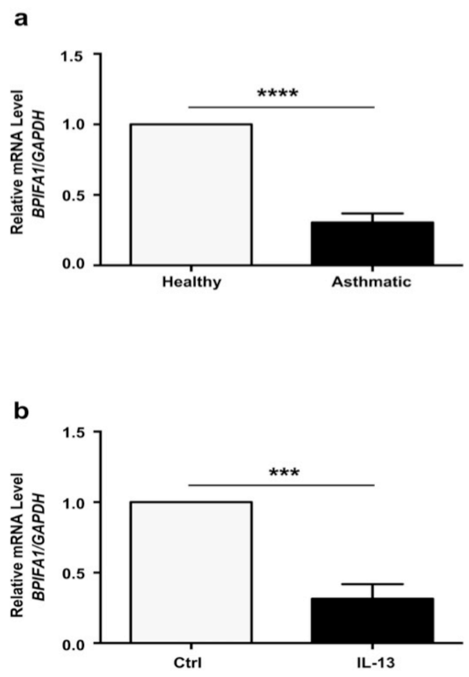


**Supplementary Figure 3. BPIFA1 mRNA is decreased in asthmatic (a) and IL-13 treated (b) human bronchial epithelial cultures (HBECs).** Total RNA from normal and asthmatic HBECs was extracted and mRNA levels of *BPIFA1* were measured by qRT-PCR (n=3/group). Data are mean±s.e.m and were analyzed using student's *t*-test. *** Indicates P<0.001 and **** indicates P<0.0001 different to control.

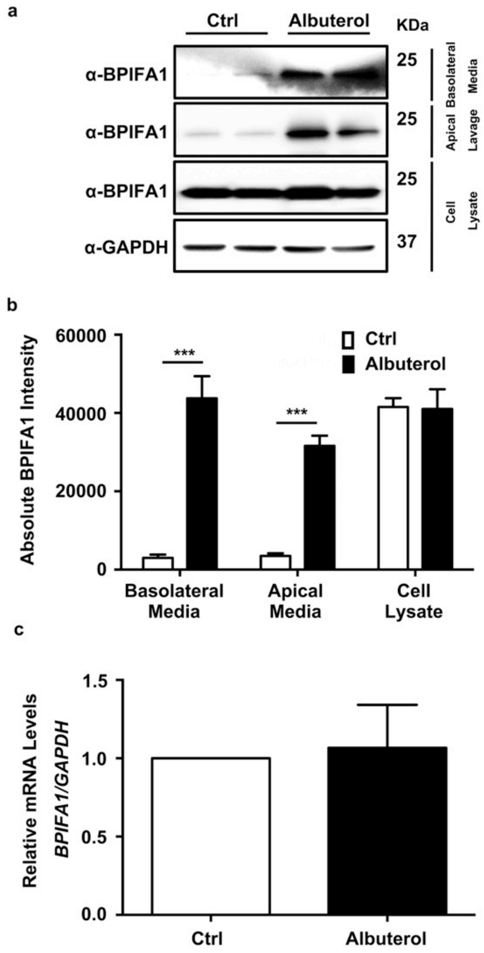


**Supplementary Figure 4. Albuterol increases BPIFA1 secretion but not *BPIFA1* expression in HBECs. (a)** Immunoblots demonstrating that BPIFA1 secretion in HBEC basolateral media and apical lavage is triggered by a 2 h exposure to albuterol (10 μM). **(b)** Mean densitometry taken from (a). **(c)** Total RNA from normal and asthmatic HBECs was extracted and mRNA levels of *BPIFA1* were quantified by qRT-PCR (n=3/group). Data in b and c are mean±s.e.m. The data were analyzed using student's *t*-test. *** indicates P<0.001 different to control.

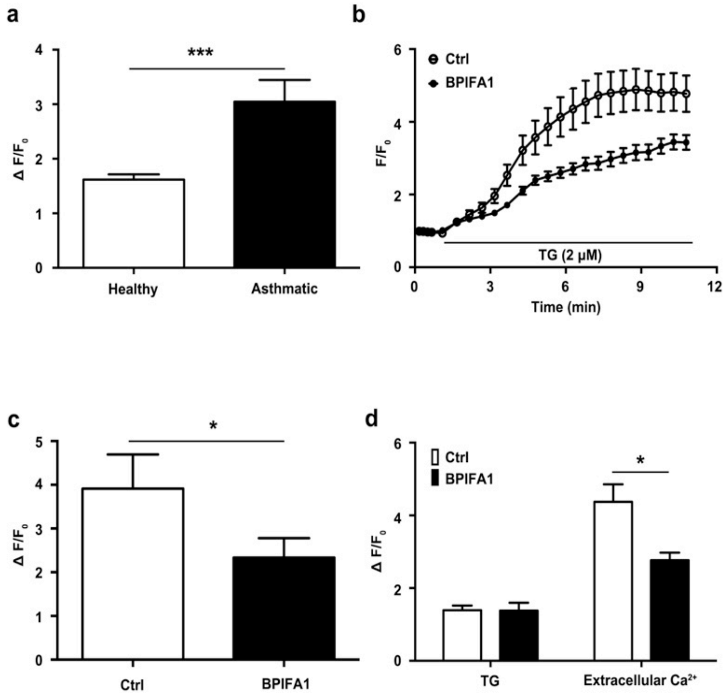


**Supplementary Figure 5. BPIFA1 suppresses thapsigargin (TG)-induced elevations in cytosolic $Ca^{2+}$ levels. (a)** Summary of peak fluorescent ratio change in ASMCs incubated with media from healthy and asthmatic HBECs, respectively (n=3/group). **(b)** Average traces of fura-2 fluorescence as an indicator of cytosolic $Ca^{2+}$ levels. ASMCs were co-incubated with fura-2 and either BPIFA1 or vehicle for 1 h. The fura-2 emission ratio was then recorded over time. **(c)** Summary of peak fluorescent ratio changes in fura-2 fluorescence with or without BPIFA1 (both n=3). **(d)** Summary of peak fluorescent ratio change in the presence of TG and extracellular $Ca^{2+}$ (n=3). Data in a, c and d are mean±s.e.m. The data were analyzed using student's t-test. * indicates P<0.05 different to control. *** indicates P<0.001 different to control.

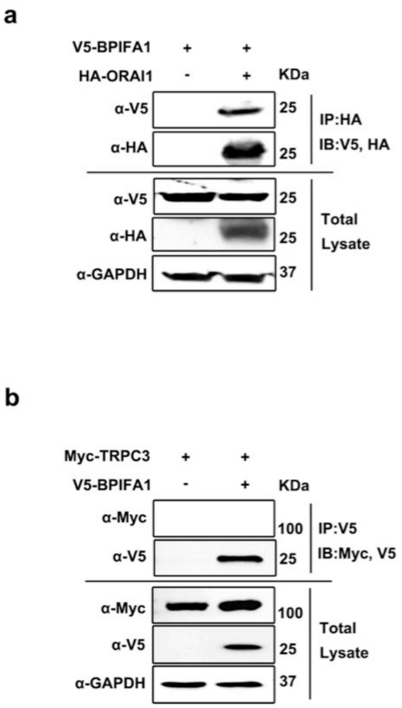


**Supplementary Figure 6. BPIFA1 does not interact with TRPC3.** Immunoprecipitation analysis was performed using cell lysates from HEK293T cells co-transfected with V5-BPIFA1 and either HA-ORAI1 **(a)** or Myc-TRPC3 **(b)**. Data are representative of 3 individual blots per condition.

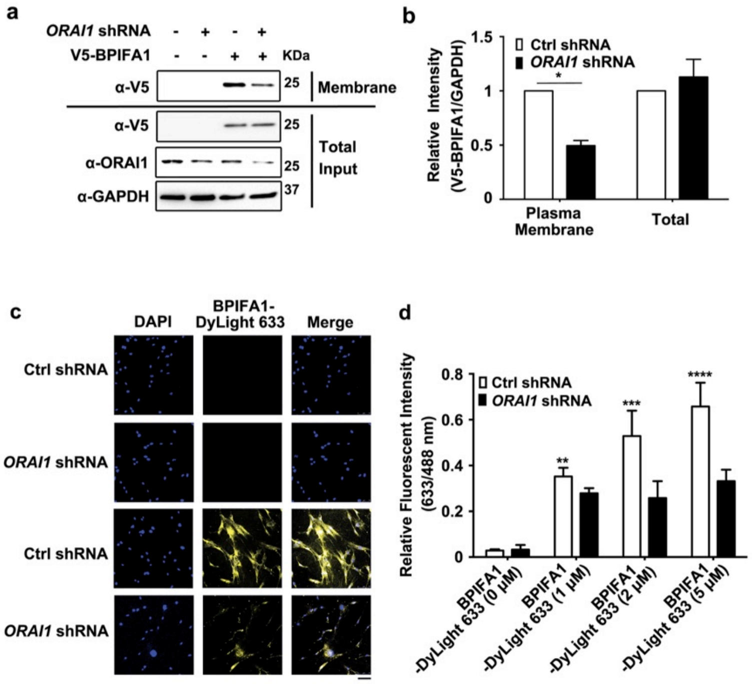


**Supplementary Figure 7. Knockdown of *ORAI1* using shRNA decreases BPIFA1 binding to ASMC plasma membrane. (a)** Human ASMCs were transfected with scrambled shRNA (control) and *ORAI1* shRNA respectively for 72 h. Cells were incubated in the presence or absence of BPIFA1, followed by surface biotinylation and immunoblot using indicated antibodies. **(b)** Intensity of immunoblots from (a) were quantified using Image J, normalized to GAPDH and expressed as relative intensity (all n=3). **(c)** Human ASMCs were transfected with scrambled control shRNA and *ORAI1* shRNA respectively for 72 h. BPIFA1 was labeled with DyLight-633 (yellow). 72 h post-transfection, cells were treated with or without BPIFA1-DyLight 633 for 1 h, then washed 5 times with ice-cold Ringer's solution. Cells were then fixed with ice-cold methanol and counter stained with DAPI (blue). Fluorescent images were taken using a Leica SP8 confocal microscope. Scale bar indicates 75 μm. (d) Bar graph showing BPIFA1-Dylight 633 binding to ASMCs as detected using a fluorescence plate reader. Cells were also stained with calcein to determine cell number per well. Relative fluorescence intensity was calculated by normalizing Dylight-633 to calcein emission. Data in b and d are mean±s.e.m. The data were analyzed using student's *t*-test in b, and two-way ANOVA followed by Sidak corrected post hoc analysis in d. * indicates $P<0.05$, ** indicates $P<0.01$, *** indicates $P<0.001$, and **** indicates $P<0.0001$ different to control.

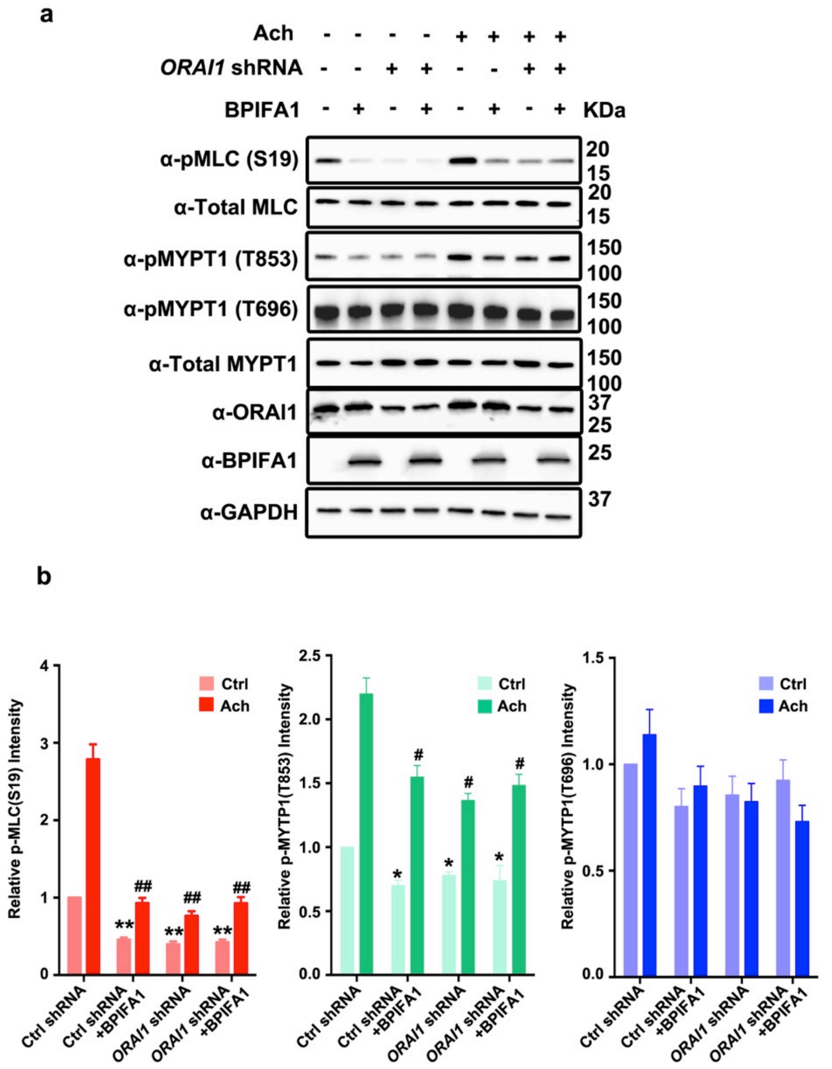


**Supplementary Figure 8. Either BPIFA1 or *ORAI1* knockdown suppresses MLC(S19) and MYTP1(T853) phosphorylation but not MYTP1 (T696) phosphorylation. (a)** Immunoblots showing phosphorylation of MLC(S19), MYTP1(T853) and MYTP1 (T696) under both basal and induced conditions. **(b)** Mean densitometry taken from (a) (n=3). Data in b are mean±s.e.m. The data were analyzed using two-way ANOVA followed by Sidak corrected post hoc analysis. * and # indicate P<0.05, ** and ## indicates P<0.01 different to control.

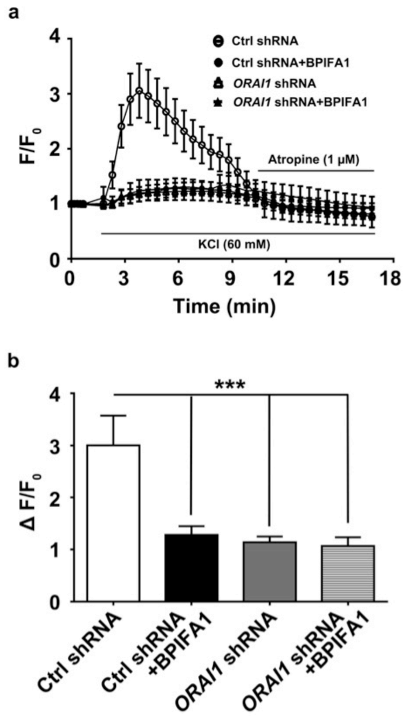


**Supplementary Figure 9. BPIFA1 or *ORAI1* knockdown blocks KCl induced $Ca^{2+}$ influx.** **(a)** Representative traces of $Ca^{2+}$ imaging using fura-2. Human ASMCs were transfected with control and *ORAI1* shRNA respectively for 72 h. After incubating with or without 10 μM BPIFA1 for 1 h, the fura-2 emission ratio was then recorded in the presence of 60 mM KCl, and 1 μM atropine was added at indicated time point. **(b)** Summary of peak fluorescent ratio changes in the presence of TG (n=3/group). Data in b are mean±s.e.m. The data were analyzed using one-way ANOVA followed by Turkey post hoc analysis. *** indicates P<0.001 different to control.

.

a

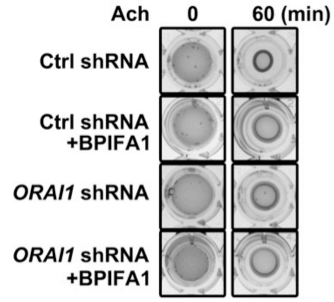


b

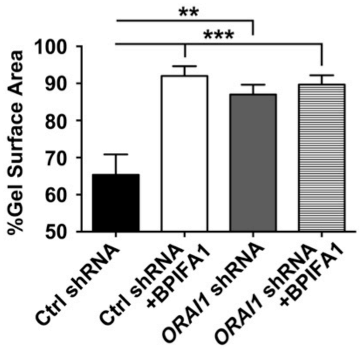


**Supplementary Figures 10.** ***ORAI1*** **knockdown decreases ASMC contractility** ***in vitro*****. (a)** Human ASMCs were transfected with control or *ORAI1* shRNA respectively for 72 h and then grown in a type I collagen matrix in 24 well plates. ASMC were incubated with BPIFA1 (10 µM) or vehicle control for 1 h and the gel contraction was performed ± Ach (100 µM). Representative images of the gel contraction assay at indicated time points. **(b)** Summary of contraction data expressed as decrease in gel surface area (%) at 60 min (n=3/group). Data in b are mean±s.e.m. The data were analyzed using one-way ANOVA followed by Turkey post hoc analysis. * indicates P<0.05 and ** indicates P<0.01 different to control.

Figure 1a

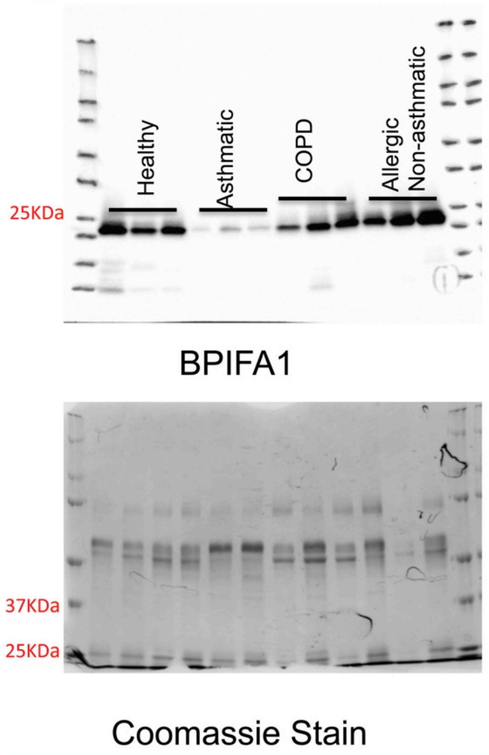


Figure 2e

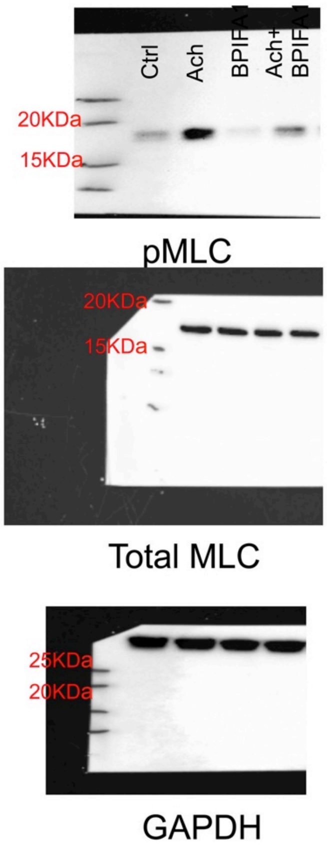


Figure 3a

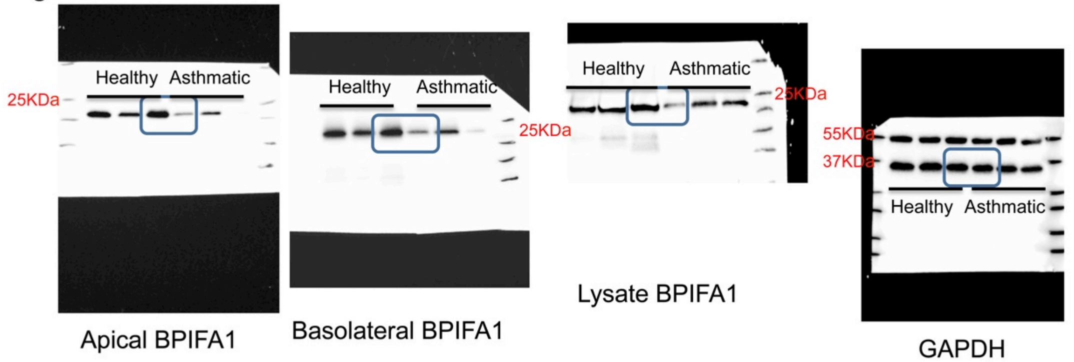


## Supplementary Figure 11

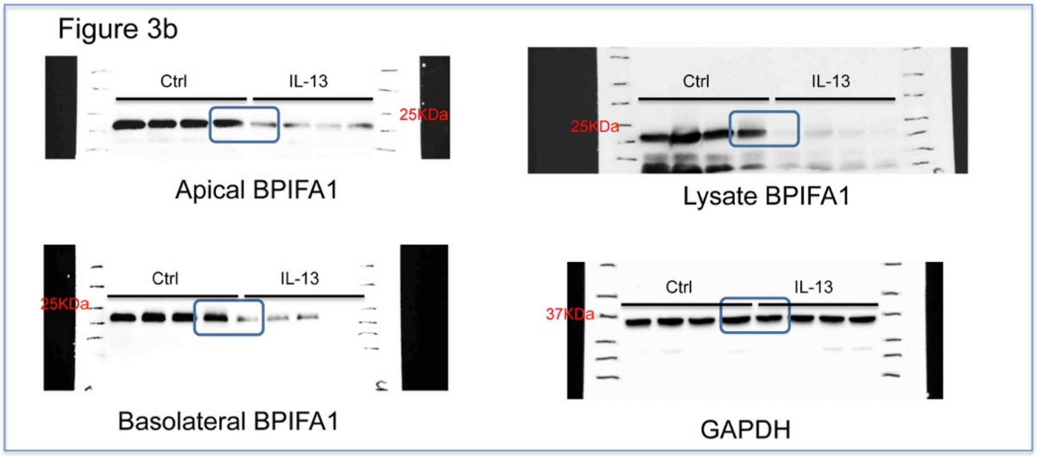


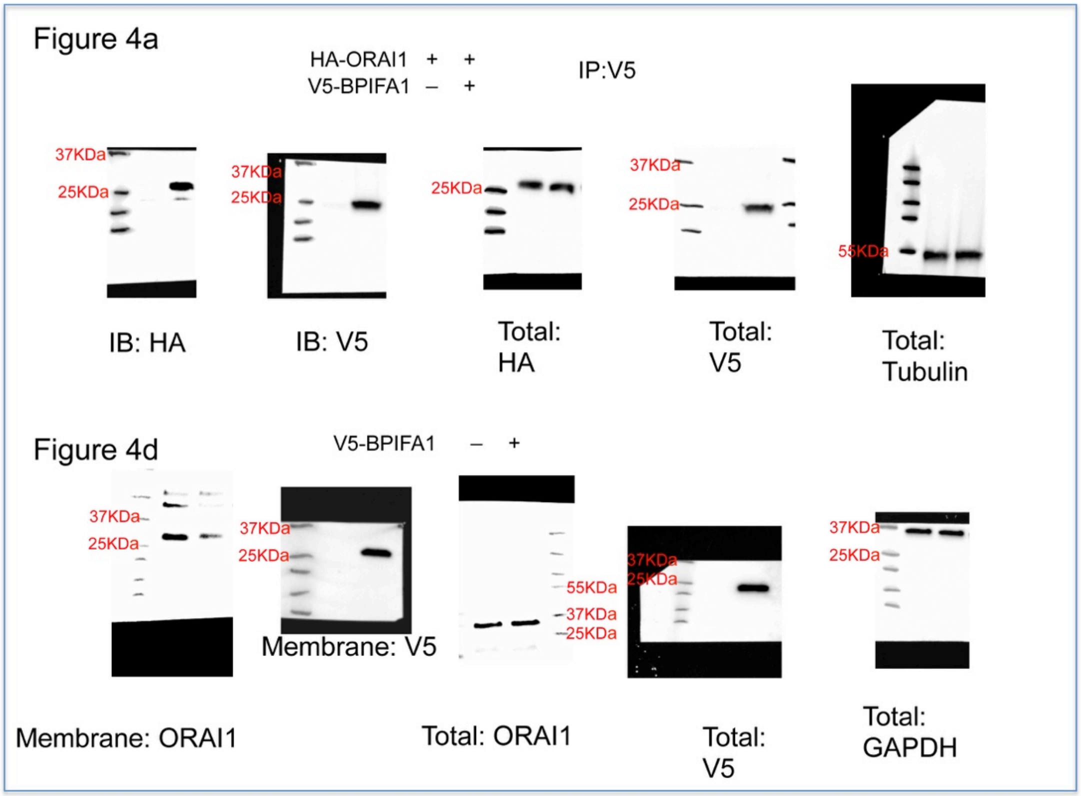


**Supplementary Figure 11**

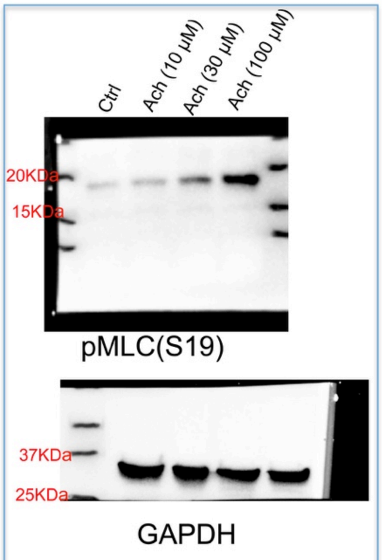


Supplementary Figure 2a

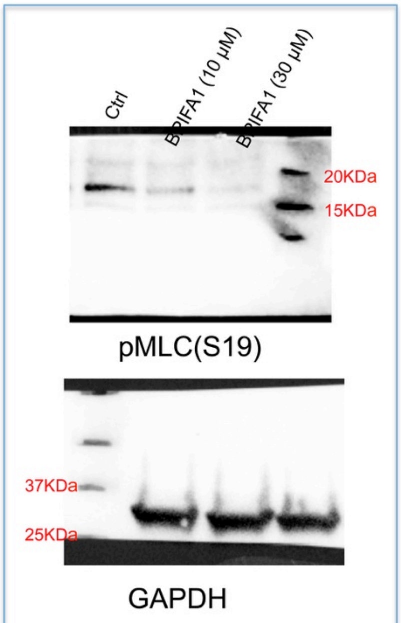


Supplementary Figure 2c

Supplementary Figure 4a

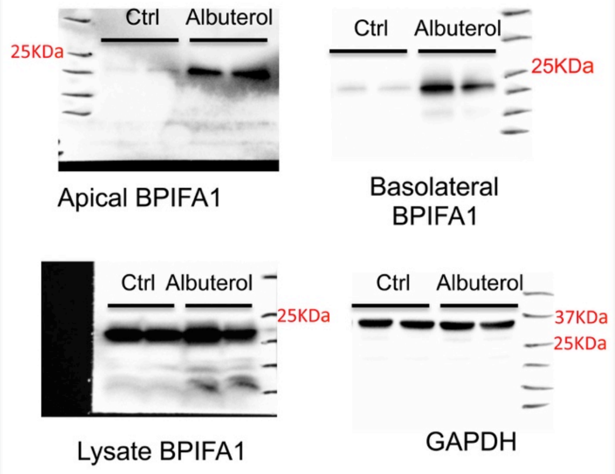


**Supplementary Figure 11**

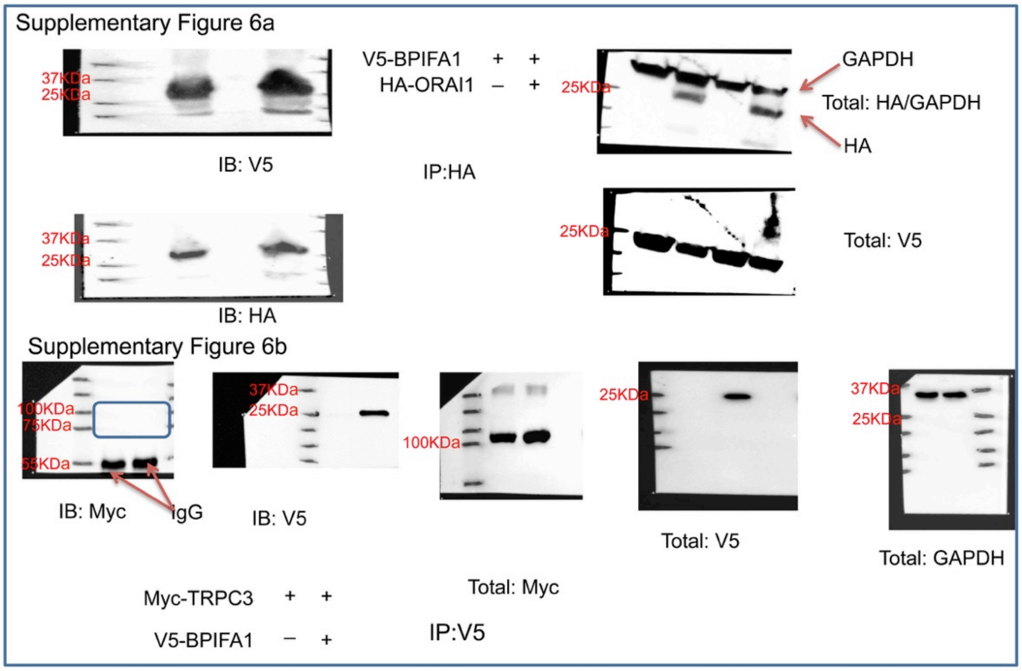


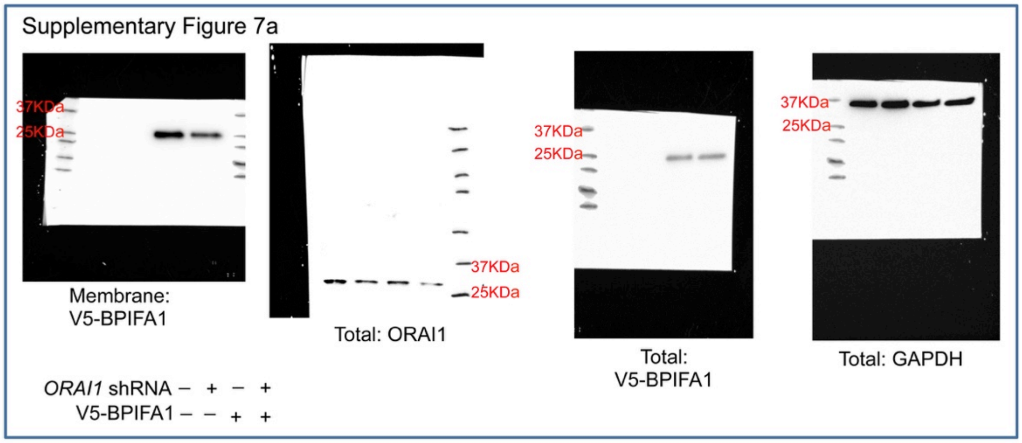


# Supplementary Figure 11

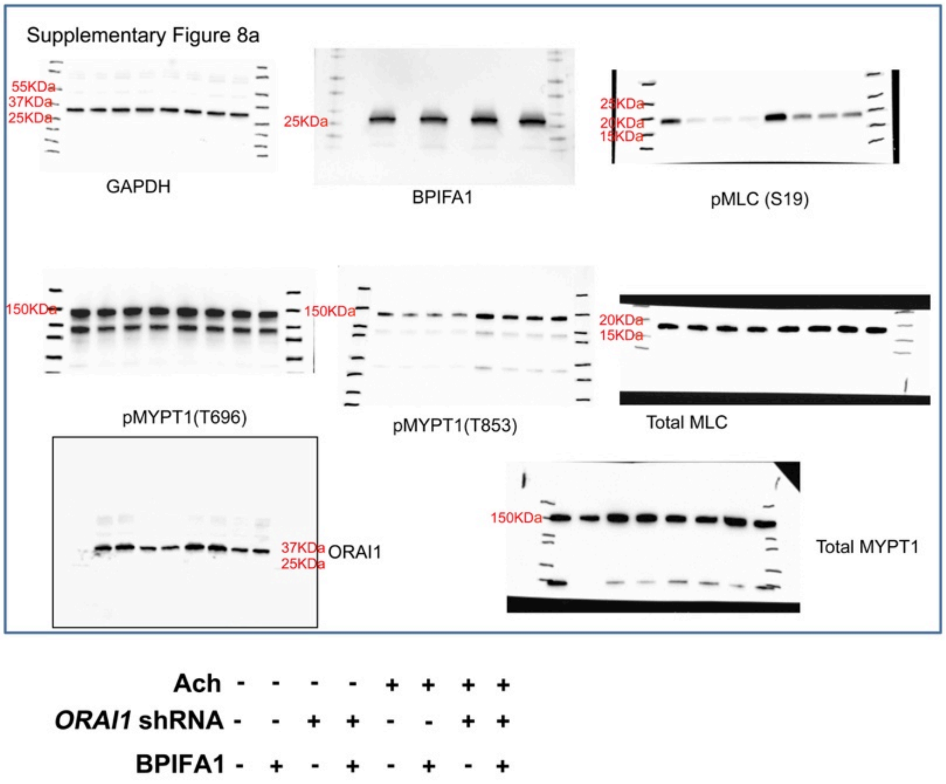


**Supplementary Figure 11**

**Supplementary Figures 11.** Original un-cropped images of all Western blots shown in the Figures and Supplementary Figures. We used protein ladders (Bio-Rad #161-0374) to identify the molecular weight of the proteins of interest.

| Donor Disease Status | Age | Gender | FVC | $FEV_1$ | FVC %PRED | $FEV_1$ %PRED |
|---|---|---|---|---|---|---|
| Nonasthmatic /nonallergic/nonsmoker | 21 | M | 6.48 | 5.25 | 117 | 110 |
| Nonasthmatic /nonallergic/nonsmoker | 23 | F | 4.2 | 3.25 | 117 | 105 |
| Nonasthmatic /nonallergic/nonsmoker | 20 | F | 3.64 | 3.08 | 84 | 84 |
| Nonasthmatic /nonallergic/nonsmoker | 23 | M | 6.05 | 4.77 | 106 | 96 |
| Nonasthmatic /nonallergic/nonsmoker | 28 | F | 4.6 | 3.44 | 115 | 102 |
| Nonasthmatic /nonallergic/nonsmoker | 20 | M | 5.15 | 4.1 | 107 | 99 |
| | | | | | | |
| Asthmatic/nonsmoker | 22 | F | 3.76 | 2.93 | 101 | 91 |
| Asthmatic/allergic | 21 | F | 4.27 | 3.26 | 103 | 90 |
| Asthmatic/allergic | 36 | M | 5.85 | 4.66 | 102 | 102 |
| Asthmatic/allergic | 31 | F | 3.52 | 2.8 | 105 | 98 |
| Asthmatic/allergic | 21 | F | 4.84 | 3.65 | 122 | 106 |
| Asthmatic/allergic | 26 | F | 4.74 | 3.32 | 119 | 96 |
| | | | | | | |
| COPD | 62 | F | 2.01 | 1.14 | 74 | 54 |
| COPD | 46 | F | 3.82 | 2.92 | 101 | 96 |
| COPD | 67 | M | 3.92 | 1.82 | 48 | 48 |
| COPD | 70 | F | 2.06 | 1.12 | 72 | 51 |
| COPD | 52 | M | 3.4 | 1.66 | 72 | 45 |
| COPD | 58 | M | 4.21 | 1.2 | 84 | 32 |
| | | | | | | |
| Healthy/allergic | 37 | F | 3.53 | 3.07 | 103 | 108 |
| Healthy/allergic | 25 | F | 4.23 | 3.47 | 115 | 111 |
| Healthy/allergic | 22 | F | 2.89 | 2.59 | 94 | 97 |
| Healthy/allergic | 28 | M | 4.89 | 4.09 | 87 | 87 |
| Healthy/allergic | 27 | M | 4.89 | 3.86 | 98 | 92 |
| Healthy/allergic | 30 | M | 5.02 | 4.18 | 105 | 104 |

**Supplementary Table 1. Demographic information of sputum donors in Figure 1.**

| Donor disease status | Age | Gender |
|---|---|---|
| Healthy/nonasthmatic/non-smoker | 19 | M |
| Healthy/nonasthmatic/non-smoker | 45 | M |
| Healthy/nonasthmatic/non-smoker | 49 | M |
| | | |
| Asthmatic/smoker | 50 | F |
| Asthmatic/smoker | 25 | M |
| Asthmatic/smoker | 38 | F |

**Supplementary Table 2. Demographic information of lung donors pertinent to Figures 3(a), (c) and Supplementary Figure 3(a).**
